# Supplementary material for: A secretory protein neudesin regulates splenic red pulp macrophages in erythrophagocytosis and iron recycling
Source: Commun Biol. 2024 Jan 25;7:129. doi: 10.1038/s42003-024-05802-9 (PMC10811329; doi:10.1038/s42003-024-05802-9)
Supplement: Supplementary file 2 — Supplementary Information [file 42003_2024_5802_MOESM2_ESM.pdf]

Supplementary Information

Supplementary Figures

Supplementary Figure 1: Erythropoiesis were unaltered in WT and *neudesin* KO mice.

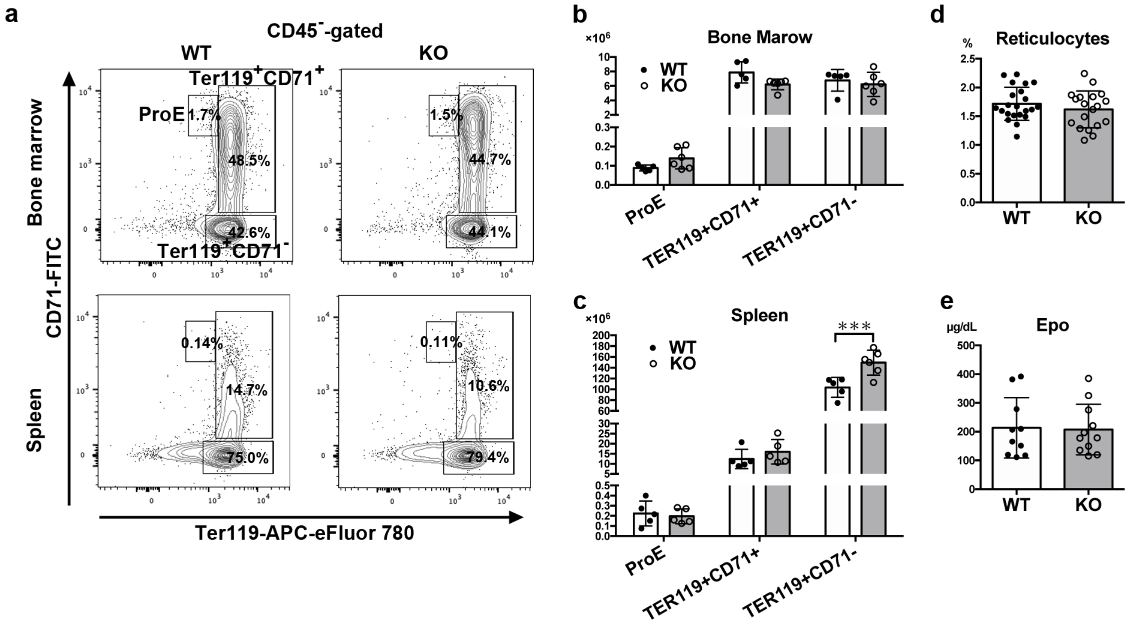

a Bone marrow cells (upper panels) and splenocytes (lower panels) from 8-week-old WT and *neudesin* KO mice were stained with anti-TER119 and anti-CD71 mAbs. Representative flow cytometry plots show populations of TER119^{lo}CD71^{hi} pro-erythroblasts (ProE), Ter119^{hi}CD71^{+} early erythroblasts, and TER119^{hi}CD71^{-} late erythroblasts and mature erythrocytes. **b, c** The chart shows the number of TER119^{lo}CD71^{hi} ProE, Ter119^{hi}CD71^{+} early erythroblasts, and TER119^{hi}CD71^{-} late erythroblasts and mature erythrocytes in bone marrow cells **b** and splenocytes **c** from 8-week-old WT and *neudesin* KO mice. **d** The chart shows the ratios of acridine orange-positive reticulocytes in peripheral blood from 8-week-old WT and *neudesin* KO mice. **e** The chart shows the concentrations of serum erythropoietin from 8-week-old WT and *neudesin* KO mice. Each symbol represents an individual mouse. Data are shown as means ± SD of three or more experiments. ***$P < 0.001$ by unpaired t-test.

Supplementary Figure 2: Single dose administration of recombinant Neudesin protein did not rescue the phenotypes of in *neudesin* KO mice.

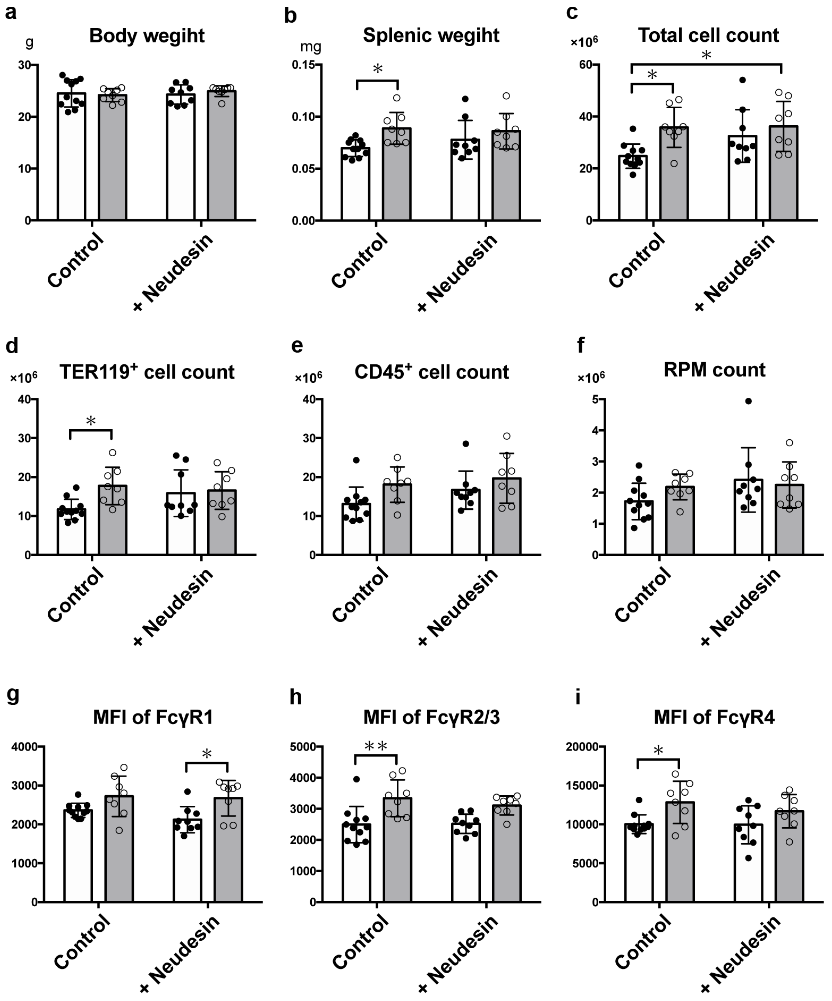

8-week-old WT and *neudesin* KO mice were intravenously administrated of 100 μL PBS (Control), or 1 μg recombinant Neudesin proteins (+ Neudesin). Body weights **a**, splenic weights **b**, and cell numbers of splenocytes **c** were measured 24 h after administration. **d-i** 24 hours after administration of recombinant Neudesin proteins, splenocyte suspensions were assessed by flow cytometric analysis, and cell numbers of $TER119^+$ red blood cells **d**, $CD45^+$ white blood cells **e**, and $F4/80^{hi}$ RPMs in the spleen were counted. The MFI of surface FcγRs on RPMs were also measured by flow cytometry. Each symbol represents an individual mouse. Data are shown as means ± SD of three or more experiments. **P* < 0.05, and ***P* < 0.01 by a 2-way analysis of variance followed by Sidak's post-test.

Supplementary Figure 3: Administration of three consecutive days with recombinant Neudesin protein did not rescue the phenotypes of in *neudesin* KO mice.

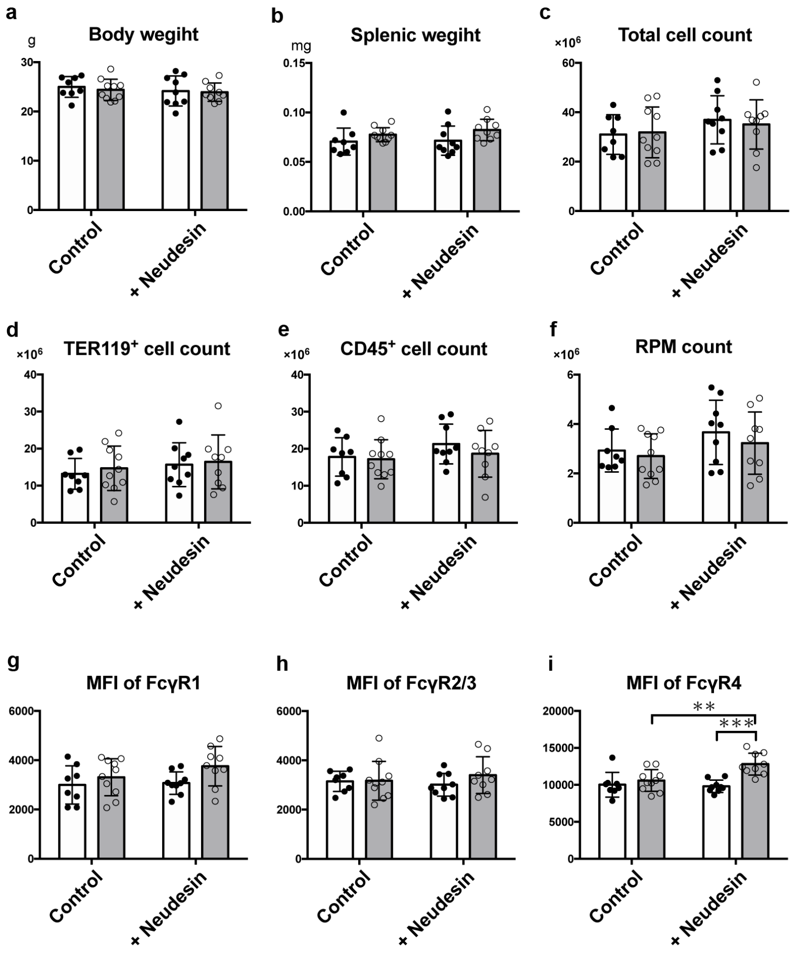

8-week-old WT and *neudesin* KO mice were intravenously administrated of 100 μL PBS (Control), or 1 μg recombinant Neudesin proteins (+ Neudesin) for 3 consecutive days. Body weights **a**, splenic weights **b**, and cell numbers of splenocytes **c** were measured 24 h after administration. **d-i** 24 hours after administration of recombinant Neudesin proteins, splenocyte suspensions were assessed by flow cytometric analysis, and cell numbers of $TER119^+$ red blood cells **d**, $CD45^+$ white blood cells **e**, and $F4/80^{hi}$ RPMs in the spleen were counted. The MFI of surface FcγRs on RPMs were also measured by flow cytometry. Each symbol represents an individual mouse. Data are shown as means ± SD of three or more experiments. $^{**}P < 0.01$, and $^{***}P < 0.001$ by a 2-way analysis of variance followed by Sidak's post-test.

Supplementary Figure 4: Gating strategies of splenocytes for TER119+ erythrocytes, CD45+ lymphocytes, and RPMs.

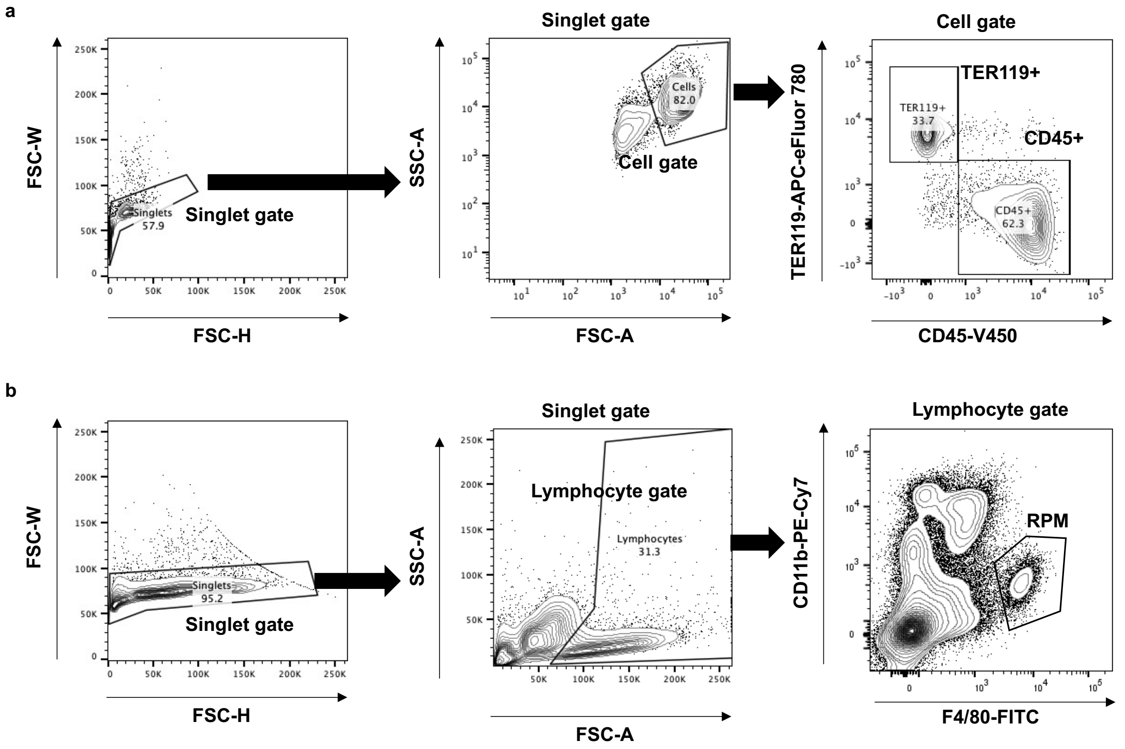

a, b Representative flow cytometry plots from splenocytes of 8-week-old WT mice to identify **a** TER119+ erythrocytes and CD45+ lymphocytes, and **b** red pulp macrophages (RPMs).

.

Supplementary tables

Supplementary Table 1: Primer sequences used for gene expression analysis by quantitative RT-PCR.

Gene	**Forward**	**Reverse**
Adgre1	5'-CATCAGCCATGTGGGTACAG-3'	5'-AGTGTAGGAATCCCGCAATG-3'
Cd163	5'-TCCACACGTCCAGAACAGTC-3'	5'-CCTTGGAAACAGAGACAGGC-3'
Fcgr1	5'-TCCTCAATGCCAAGTGACCC-3'	5'-GAATGGCGACCTCCGAATCT-3'
Fcgr2b	5'-CAGTCCAAGCCTGTCACCAT-3'	5'-TCCCTGTGATCAGGGTTTCC-3'
Fcgr3	5'-TGCACACTCTGGAAGCCAAT-3'	5'-TCCCTTCGCACATCAGTGTC-3'
Fcgr4	5'-CGATCAGTGACCCAGTGCAA-3'	5'-CCGTACAGGTCTGTTTTGCC-3'
Hmox1	5'-GAATCGAGCAGAACCAGCCT-3'	5'-CTCAGCATTCTCGGCTTGGA-3'
neudesin	5'-ACTTGGCAGTGAAGGGAGTG-3'	5'-CCGTGAGACCAGTAGTGTCG-3'
Slc40a1	5'-TAAAGTGGCCCAGACGTCAG-3'	5'-AGCAGACAGTAAGGACCCATC-3'
Spic	5'-TCCGCAACCCAAGACTCTTCAA-3'	5'-GGGTTCTCTGTGGGTGACATTCCAT-3'
18S ribosomal RNA	5'-CCGGAATCGAACCCTGATT-3'	5'-CGAACGTCTGCCCTATCAACTT-3'

Supplementary Table 2: List of antibodies and other reagents for flow cytometry and western blotting.

	Clone	Fluorochrome	Source
**Antibody**			
TER-119	TER-119	APC-eFluor 780	Thermo Fisher Scientific
CD45	30-F11	V450	BD Biosciences
Anti-mouse IgG	Chicken polyclonal	Alexa Fluor 488	Thermo Fisher Scientific
Anti-mouse IgM	Chicken polyclonal	Alexa Fluor 488	Thermo Fisher Scientific
F4/80	BM8	FITC, APC-Cy7	BioLegend
CD11b	M1/70	PE-Cy7	BD Biosciences
B220	RA3-6B2	FITC	BD Biosciences
CD4	RM4-5	PE	BioLegend
CD8	53-6.7	PE-Cy5	BD Biosciences
CD11c	N418	APC-Cy7	BioLegend
Gr-1	RB6-8C5	PE	BioLegend
NK1.1	PK136	FITC	BD Biosciences
VCAM1	M/K-2	PE	Beckman Coulter
Stabilin-2	#34-2	FITC	MBL
Tim-4	RMT4-54	PE	BioLegend
Axl	MAXL8DS	PE	Thermo Fisher Scientific
FcγR1	X54-5/7.1	PE	BioLegend
FcγR2/3	2.4G2	FITC	BD Biosciences
FcγR4	9E9	PE	BD Biosciences
SIRPα	P84	PerCP-Cy5.5	BioLegend
CD163	TNKUPJ	PE	Thermo Fisher Scientific
CD206	C068C2	APC	BioLegend
CD71	R17217	FITC	BioLegend
Phospho-ERK	D13.14.4E	-	Cell signaling
ERK	137F5	-	Cell signaling
Phospho-AKT	D9E	-	Cell signaling
AKT	C67E7	-	Cell signaling
Phospho-CREB	87G3	-	Cell signaling
CREB	46H2	-	Cell signaling
β-Actin	Polyclonal	-	Proteintech
**Others**			
Annexin V	-	FITC	BD Biosciences
Acridin orange	-	-	Nacalai Tesque
Streptavidin	-	Alexa Fluor 594	Thermo Fisher Scientific

Supplementary Methods

Reticulocyte count

Peripheral blood samples were harvested in EDTA-coated microtubes (Health Wave Japan, Tokyo, Japan), stained with 0.05% acridine orange in PBS, and analyzed using a FACSAriaIII and FlowJo software (BD Biosciences, Franklin Lakes, NJ, USA).

ELISA for serum erythropoietin

Serum concentration of erythropoietin was measured with Mouse Erythropoietin Quantikine ELISA Kit (R&D systems, Minneapolis, MN, USA)
