# Supplementary material for: A secretory protein neudesin regulates splenic red pulp macrophages in erythrophagocytosis and iron recycling
Source: Commun Biol. 2024 Jan 25;7:129. doi: 10.1038/s42003-024-05802-9 (PMC10811329; doi:10.1038/s42003-024-05802-9)
Supplement: Supplementary file 3 — Description of Supplementary Materials [file 42003_2024_5802_MOESM3_ESM.docx]

**Description of Additional Supplementary Files**

**File name:** Supplementary Data

**Description:** The source data underlying the graphs and charts in the paper
